# The diagnostic performance of ultrafast MRI to differentiate benign from malignant breast lesions: a systematic review and meta-analysis

**DOI:** 10.1007/s00330-024-10690-y

**Published:** 2024-03-21

**Authors:** Yoav Amitai, Vivianne A. R. Freitas, Orit Golan, Rivka Kessner, Tamar Shalmon, Rina Neeman, Michal Mauda-Havakuk, Diego Mercer, Miri Sklair-Levy, Tehillah S. Menes

**Affiliations:** 1 Department of Medical Imaging, Tel Aviv University, Sackler School of Medicine, Sourasky Medical Center, Weizmann 6, 6423906 Tel Aviv-Yafo, Israel; 2 Joint Department of Medical Imaging – University Health Network, Sinai Health System, Women’s College Hospital, University of Toronto, 610 University Avenue – M5G 2M9, Toronto, Ontario Canada; 3 Department of Medical Imaging, Sackler School of Medicine, Chaim Sheba Medical Center, Tel Aviv University, Tel Hashomer, Derech Shiba 2, 52621 Ramat-Gan, Israel; 4 Department of Surgery, Sackler School of Medicine, Chaim Sheba Medical Center, Tel Aviv University, Tel Hashomer, Derech Shiba 2, 52621 Ramat-Gan, Israel

**Keywords:** Breast cancer, Magnetic resonance imaging, Ultrafast, Kinetics, Artificial intelligence

## Abstract

**Objectives:**

To assess the diagnostic performance of ultrafast magnetic resonance imaging (UF-DCE MRI) in differentiating benign from malignant breast lesions.

**Materials and methods:**

A comprehensive search was conducted until September 1, 2023, in Medline, Embase, and Cochrane databases. Clinical studies evaluating the diagnostic performance of UF-DCE MRI in breast lesion stratification were screened and included in the meta-analysis. Pooled summary estimates for sensitivity, specificity, diagnostic odds ratio (DOR), and hierarchic summary operating characteristics (SROC) curves were pooled under the random-effects model. Publication bias and heterogeneity between studies were calculated.

**Results:**

A final set of 16 studies analyzing 2090 lesions met the inclusion criteria and were incorporated into the meta-analysis. Using UF-DCE MRI kinetic parameters, the pooled sensitivity, specificity, DOR, and area under the curve (AUC) for differentiating benign from malignant breast lesions were 83% (95% CI 79–88%), 77% (95% CI 72–83%), 18.9 (95% CI 13.7–26.2), and 0.876 (95% CI 0.83–0.887), respectively. We found no significant difference in diagnostic accuracy between the two main UF-DCE MRI kinetic parameters, maximum slope (MS) and time to enhancement (TTE). DOR and SROC exhibited low heterogeneity across the included studies. No evidence of publication bias was identified (*p* = 0.585).

**Conclusions:**

UF-DCE MRI as a stand-alone technique has high accuracy in discriminating benign from malignant breast lesions.

**Clinical relevance statement:**

UF-DCE MRI has the potential to obtain kinetic information and stratify breast lesions accurately while decreasing scan times, which may offer significant benefit to patients.

**Key Points:**

• *Ultrafast breast MRI is a novel technique which captures kinetic information with very high temporal resolution.*

• *The kinetic parameters of ultrafast breast MRI demonstrate a high level of accuracy in distinguishing between benign and malignant breast lesions.*

• *There is no significant difference in accuracy between maximum slope and time to enhancement kinetic parameters.*

**Supplementary Information:**

The online version contains supplementary material available at 10.1007/s00330-024-10690-y.

## Introduction

Dynamic contrast-enhanced magnetic resonance imaging (DCE-MRI) is widely regarded as an excellent tool for evaluating inconclusive breast lesions, thanks to its high sensitivity and reliable negative predictive value in excluding malignancy [1–6]. However, a comprehensive breast MRI study protocol can be time-consuming. As a result, various efforts have been made to reduce scan times, aiming to make breast MRI more affordable and accessible. One such example is abbreviated breast MRI (AB-MRI), which substantially shortens the scan duration and necessitates only one post-contrast scan [7]. Several works have demonstrated the noninferiority of AB-MRI in cancer detection when compared to DCE-MRI [8–11]. However, one potential limitation of AB-MRI is the absence of kinetic information.

Ultrafast MRI (UF-DCE MRI) is a relatively novel technique which captures kinetic information within the first minute with very high temporal resolution (typically less than 7 s). Instead of relying on the conventional washout characteristics, the technique enables analysis of early contrast wash-in curves [12, 13]. Since UF-DCE MRI requires very high temporal resolution with an acceptable spatial resolution, several advanced MRI techniques have been used including view-sharing (VS), parallel imaging (PI), and compressed sensing (CS).

UF-MRI kinetic parameters, proposed as alternatives to those derived from conventional DCE-MRI, capture common pathophysiological processes in breast cancer, such as rapid contrast leakage [14] and tumor-associated vascular shunting [15]. Consequently, malignant lesions tend to exhibit more rapid enhancement on UF-MRI when compared to benign ones.

**Fig. 1:**
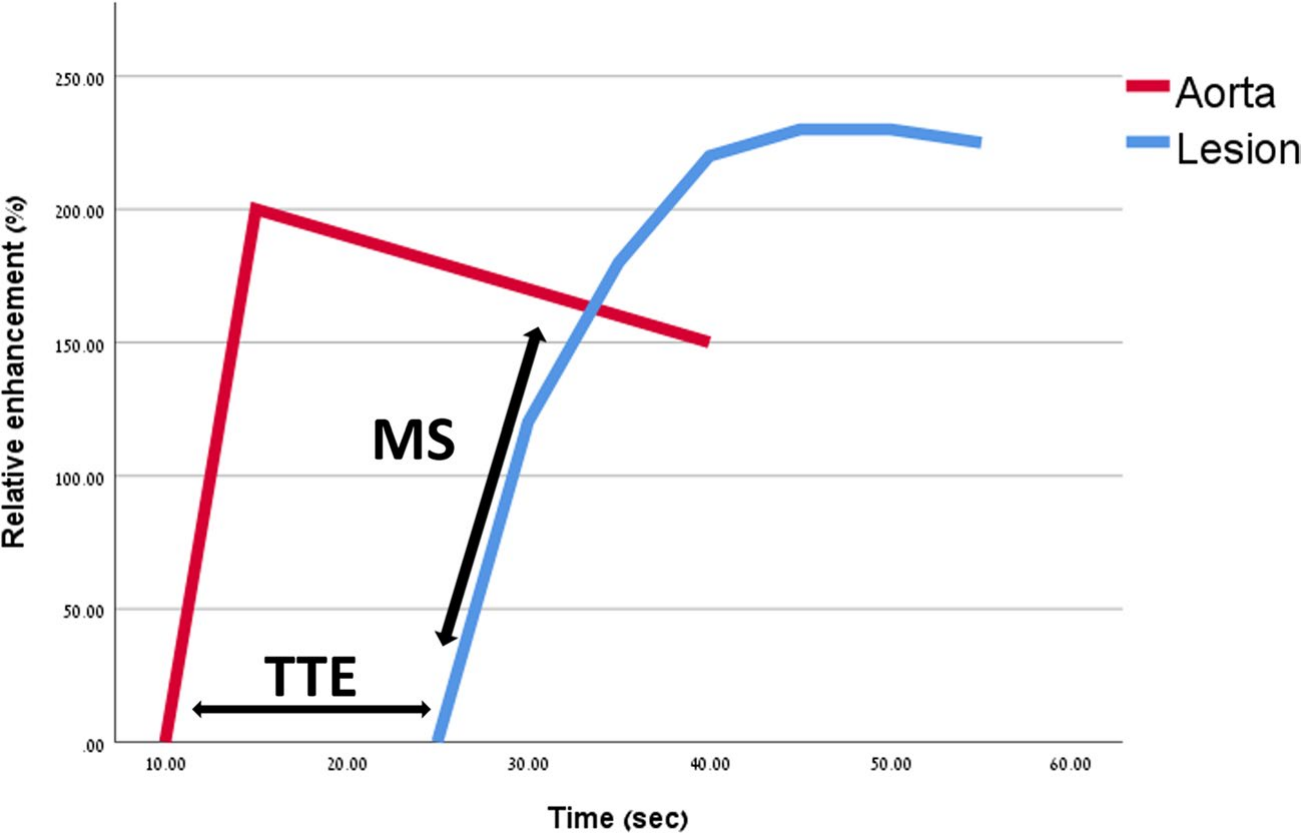
Graphic illustration of MS and TTE. MS is defined as the steepest part of the lesion’s enhancing curve (%/s). TTE is calculated by the time point in which the lesion starts to enhance minus the time point where the aorta starts to enhance (s). MS, maximun slope. TTE, time to enhancement

Maximum slope (MS), suggested by Mann et al in 2014 [16], is determined by drawing a tangent along the steepest part of the enhancing curve within the first minute, and calculating the relative enhancement percentage change divided by seconds (%/s). Time to enhancement (TTE), proposed by Mus et al [17], is calculated by the time point in which the lesion starts to enhance minus the time point where the aorta starts to enhance on the maximum intensity projection images (MIP). Figure 1 shows an illustration of MS and TTE.

Less frequently utilized kinetic parameters have been proposed, all grounded in the same fundamental pathophysiological mechanisms, showing variable success in differentiating benign from malignant processes. Examples include bolus arrival time (BAT) which closely resembles TTE [18], the time interval between arterial and venous visualization (AVI) [19], and the kinetic area under the curve (KAUC) [20].

While numerous studies have explored the diagnostic performance of UF-DCE MRI, a comprehensive quantitative review to consolidate the existing body of research has not been conducted until now. The goal of this systematic review and meta-analysis was to assess the diagnostic performance of UF-DCE MRI in differentiating benign from malignant breast lesions.

## Materials and methods

Our protocol was constructed and registered at the Research Registry (Researchregistry1731, researtchregistry.com). Our work was performed by following the updated Preferred Reporting Items for Systematic Reviews and Meta-Analysis guidelines (PRISMA 2020 [21]). A previous paper describing the approach to diagnostic test accuracy (DTA) meta-analysis in R software was used as a reference [22].

### Literature search

A comprehensive search of Medline, Embase, and Cochrane was conducted for relevant articles published from each database’s inception up to September 1, 2023. Both controlled vocabulary terms and text words were used. No age restrictions were applied. Details of the search strategy are included in the Supplemental materials. Reference lists of included studies were manually searched to identify additional relevant studies. The search strategy is shown in Table S1.

### Eligibility criteria

Studies were included if they met the following criteria: (a) The full text was available in English. (b) Published in a peer-reviewed journal (abstracts and conference presentations excluded). (c) MRI acquisition data was available. (d) The purpose of the study was to assess the diagnostic performance of UF-DCE MRI in differentiating benign from malignant breast lesions. Only studies using UF-MRI kinetic parameters exclusively to stratify lesions were used in this work. Studies using artificial intelligence (AI) methods, such as convolutional neural networks or radiomics, were excluded. (e) The study reported sufficient data to calculate the number of true-positive (TP), false-positive (FP), true-negative (TN), and false-negative (FN). If these values could not be obtained, the study was excluded.

### Study selection

We executed a strategy that encompassed three distinct phases.

In phase 1, the results of the literature search were imported into reference manager software (EPPI Reviewer 6, University of London, England) for independent title and abstract review by multiple investigators (Y.A., T.M., O.G., and R.K.). Prior to commencing the independent review of titles and abstracts, reviewers conducted a preliminary screening of 20 studies in duplicate, aimed at enhancing consistency among the reviewers.

In phase 2, the full-text articles from potentially eligible sources were retrieved, and three investigators (Y.A., T.M., O.G., and R.K.) independently assessed them for inclusion. Any discrepancies were resolved by consensus.

In phase 3, three reviewers (Y.A., T.M., and O.G.) independently assessed the risk of bias using Quality Assessment of Diagnostic Accuracy Studies-2 (QUADAS-2 [23]). Any discrepancies were again resolved by consensus.

### Data extraction

Data were extracted into a spreadsheet program (Microsoft Excel 2021; Microsoft, Redmond, USA) independently by multiple investigators (Y.A., T.M., O.G., and R.K.). Prior to commencing the data extraction, reviewers extracted data from three studies in duplicate, aimed at enhancing consistency among the reviewers. Any discrepancies were resolved by consensus.

For studies that used multiple readers, readers’ data was averaged for analysis [24]. For the construction of the main contingency table, we used the model in each study that achieved the best results using only UF-DCE MRI criteria, either a single UF-MRI kinetic parameter or a combination of UF-DCE MRI parameters. When a dataset was used for multiple studies, we extracted values from only one of the studies.

The following data were extracted: title of study, first author, country of first author, journal of publication, study design, patient demographics (mean age, indication for imaging, sample size BIRADS cutoff for inclusion if available), number of cancer cases, cancer definition, reference standard used (histopathology vs imaging follow-up), MRI parameters (MRI system and magnetic field strength, temporal resolution, slice thickness, acceleration method, injection speed), UF-DCE MRI parameters (kinetic parameters used, a priori vs post hoc cutoff used to stratify lesions), number of readers for each lesion, and 2 × 2 contingency table (FP, FN, TP, TN).

### Quality assessment

Independent quality assessment of included studies was performed using the revised tool for QUADAS-2 [23]. Three investigators (Y.A., T.M., and O.G.) assessed all studies for the following criteria: patient selection, index test, reference standard, flow, and timing. The following criteria were defined as being high risk for bias: consecutive patient selection was not used (patient selection 1), inclusion limited to a particular group of patients, e.g., those who had undergone surgery or individuals with non-mass enhancement (NME) lesions (patient selection 2), radiologists were not blinded to previous clinical or imaging tests (index test), and the histopathological testing was not used for all lesions (reference standard and flow and timing). Any discrepancies were resolved by consensus.

### Outcomes

The primary outcome was to estimate the diagnostic test accuracy of UF-DCE MRI in differentiating benign from malignant lesions. In the primary analysis, we assessed diagnostic accuracy using the most accurate model from each study. Additionally, in a separate analysis, we evaluated the diagnostic accuracy of MS and TTE individually with direct comparison between them. Several covariates were used in the meta-regression models when there was sufficient variability within the data.

### Statistical analysis

Each meta-analysis was conducted to calculate the combined sensitivity, specificity, and diagnostic odds ratio (DOR), along with their respective 95% confidence intervals (CIs). Coupled forest plots and hierarchic summary operating characteristics (SROC) curves were created using the estimated model parameters. The bivariate random-effects model was used for the analysis [22, 25]. The following meta-analyses were conducted:

- The best UF-DCE MRI kinetic parameter within each study (main analysis).
- Studies assessing MS kinetic parameter individually.
- Studies assessing TTE kinetic parameter individually.

Meta-regression models were created for the main analysis. To compare diagnostic accuracy between MS and TTE, “head-to-head” meta-regression models were performed with the inclusion of all studies assessing either MS or TTE individually and the kinetic parameters as covariates.

Heterogeneity for sensitivity, specificity, and DOR was assessed using the *I*^2^ test, with values greater than 50% considered at risk for significant variability [26]. Heterogeneity for SROC curve was assessed using the correlation coefficient between sensitivity and specificity, with values larger than 0 indicating high heterogeneity [27].

The publication bias was evaluated by a funnel plot and Egger’s test.

Analysis was performed using the “mada,” “meta,” and “dmetatools” packages in R (R version 3.6.3; R Foundation for Statistical Computing). A two-sided *p* value less than 0.05 was considered statistically significant.

## Results

### Study demographics and risk of bias

**Fig. 2:**
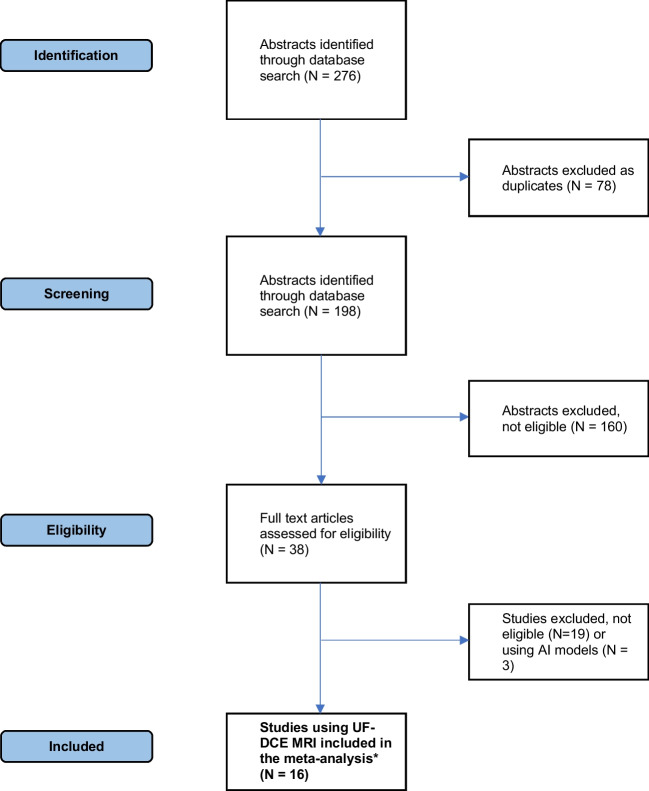
Prefered Reporting Items for Systematic Reviewes and Meta-Analysis (PRISMA) flowchart. UF-MRI, ultrafast magnetic resonance imaging. AI, artificial intelligence. *Typically, one or a combination of the following parameters is used: maximum slope, time to enhancement, bolus arrival time, arterial venous visualization, kinetic area under curve, or peak enhancement

Figure 2 illustrates the study flow diagram. In the initial stage, 198 studies underwent screening based on their titles and abstracts, and from this pool, 38 were identified as potentially meeting the eligibility criteria. Following a thorough review of their full texts, 16 studies analyzing 2090 lesions were found to meet the inclusion criteria and were subsequently incorporated into the meta-analysis [16–18, 20, 28–40]. Table 1 provides a summary of the included studies.

**Table 1:** Characteristics of included studies

Study	Year	Study design	Study population	Number of lesions	Number of cancers	MRI system	Magnetic field strength	Acceleration method	Contrast agent /injection speed (ml/s)	UF-MRI best model	Reference standard	TN	FN	TP	FP
Abe et al	2016	Retrospective	D + S	62	33	Philips	3 T	PI (SENSE)	Gadobenate dimeglumine /2	KAUC	Histology + FU	23	5	28	6
Cao et al	2023	Prospective	D	150	117	Siemens	3 T	CS (VIBE)	Gadobenate dimeglumine /2	MS + TTP + TTE + KAUC	Histology	27	11	106	6
Goto et al	2018	Retrospective	D	107	81	Siemens	3 T	VS (TWIST)	Gadoterate meglumine/2	MS + TTE	Histology + FU	20	9	72	6
Honda et al	2019	Retrospective	D	90	61	Siemens	3 T	CS (VIBE)	Gadobutrol/2	TTE	Histology	15	9	52	14
Kim et al	2022	Retrospective	Post operative	58	10	Philips	3 T	PI (SENSE)	Gadobutrol/2	TTE	Histology + FU	29	1	9	19
Lee et al	2019	Retrospective	Preoperative	202	140	GE	3 T	VS (DISCO)	Gadoterate meglumine/3	IEP	Histology + FU	56	35	105	6
Mann et al	2014	Retrospective	D	199	104	Philips	3 T	VS (TWIST)	Gadoterate meglumine/2.5	MS	Histology + FU	62	10	93	33
Mori et al	2020	Retrospective	D (NME)	77	54	Philips	3 T	PI (SENSE)	Gadobenate dimeglumine /2.5	KAUC	Histology + FU	17	10	44	6
Mus et al	2017	Retrospective	D	195	99	Siemens	3 T	VS (TWIST)	Gadoterate meglumine/3	TTE	Histology + FU	75	8	91	21
Ohashi et al	2019	Retrospective	D	139	90	Siemens	3 T	VS (KWIC)	Gadobutrol/2.5	MS	Histology + FU	31	8	81	19
Onishi et al	2020	Retrospective	D + S	125	26	GE	3 T	VS (DISCO)	Gadobutrol/2	MS + BAT	Histology	87	13	13	12
Pelissier et al	2021	Retrospective	D	210	150	Siemens	1.5 T	VS (TWIST)	Gadoterate meglumine/2	MS	Histology + FU	54	24	126	6
Peter et al	2020	Retrospective	D (masses)	83	60	Siemens	3 T	VS (TWIST)	Gadoterate meglumine/2	Peak enhancement	Histology + FU	14	9	51	9
Ramli et al	2023	Prospective	D	83	49	Siemens	3 T	VS (TWIST)	Gadoterate meglumine/2	MS	Histology	29	13	36	5
Van zeist et al	2018	Prospective	S (high risk)	201	31	Siemens	3 T	VS (TWIST)	Gadoterate meglumine/2.5	MS + TTE	Histology	139	5	26	31
Yamaguchi et al	2022	Retrospective	D	110	88	Siemens	3 T	CS (VIBE)	Gadobutrol/2.5	TTE	Histology + FU	20	32	56	2

*AI* artificial intelligence, *BAT* bolus arrival time, *CNN* convoluted neural network, *D* diagnostic, *CS* compression sensing, *FN* false negative, *FP* false positive, *FU* follow-up, *GE* General Electric, *IEP* initial enhancement phase, *KAUC* kinetic area under curve, *MS* maximum slope, *MRI* magnetic resonance imaging, *PI* parallel imaging, *S* screening, *TN* true negative, *TP* true positive, *TTE* time to enhancement, *TTP* time to peak, *VS* view sharing

Table 2S provides a risk of bias summary. Three studies were at low risk of bias [28, 37, 38], while the remaining 13 studies were at high risk or unknown risk of bias. The main sources of bias included no indication as to whether the patients were selected consecutively, radiologists unblinded to previous imaging or clinical data, and histopathology not employed as the reference standard for all lesions.

**Table 2:** Different meta-analysis models and their pooled accuracy measurements

Meta-analysis model	Number of studies	Sensitivity (95%CI**)**	Specificity (95%CI**)**	DOR (95%CI)	AUC of the ROC curve (95%CI)
Best UF-DCE MRI kinetic parameter	16	**83%** (79–88%)	**77%** (72–83%)	**18.9** (13.7–26.2)	**0.876** (0.830–0.887)
MS individually	7	**80%** (70–90%)	**77%** (68–89%)	**17.1** (11.4–25.6)	**0.865** (0.805–0.891)
TTE individually	7	**71%** (57–86%)	**80%** (69–82%)	**15.5** (8.3–28.9)	**0.857** (0.763–0.889)

*AUC* area under curve, *DOR* diagnostic odds ratio, *MS* maximum slope, *ROC* receiver operating characteristic, *TTE* time to enhancement, *UF-DCE MRI* ultrafast magnetic resonance imaging

### Data synthesis and pooling

#### Best UF-MRI kinetic parameter analysis

**Fig. 3:**
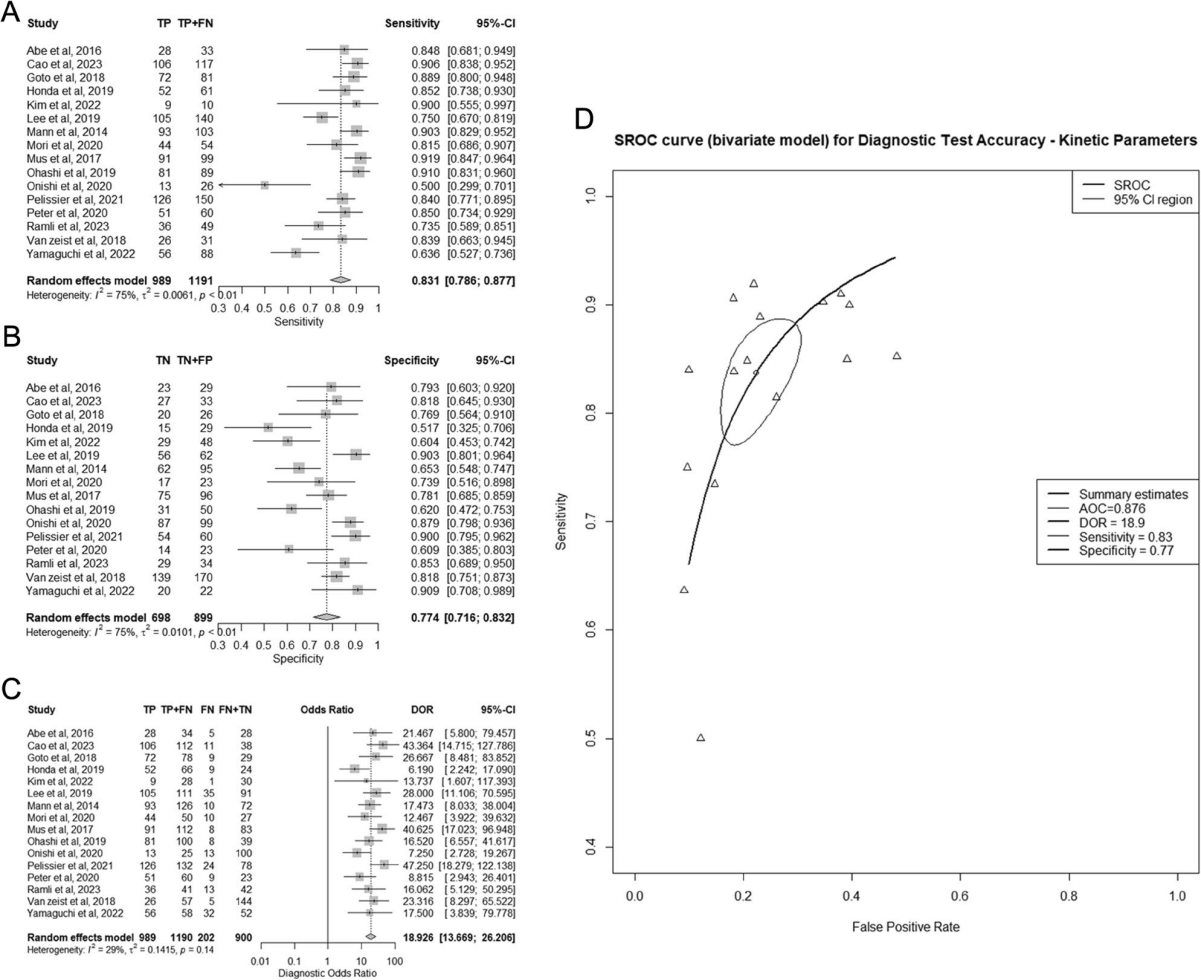
Diagnostic performance of best ultrafast magnetic resonance imaging (UF-DCE MRI) kinetic parameters for differentiating benign from malignant breast lesions. **A** Summary forest plot of sensitivity. **B** Summary forest plot of specificity. **C** Summary forest plot of diagnostic odds ratio (DOR). **D** Hierarchic summary operating characteristics (SROC) curve

Sixteen studies encompassing 2090 lesions were included in this meta-analysis [11, 16–18, 20, 28–39] (Fig. 3A–D). The best model in each study is specified in Table 1. The pooled sensitivity was 83% (95% CI 79–88%), the pooled specificity was 77% (95% CI 72–83%), the pooled DOR was 18.9 (95% CI 13.7–26.2), and the AUC of the SROC curve was 0.876 (95% CI 0.830–0.887).

Based on the *I*^2^ test, there was a high risk for substantial heterogeneity for sensitivity and specificity, and a low risk of substantial variability for DOR (Fig. 3A–C). The correlation coefficient between sensitivity and specificity was −0.62, indicating low heterogeneity of the SROC curve.

In multivariate meta-regression models (as shown in Table 3S), we observed that a lower mean age and the use of Siemens MRI systems were linked to increased sensitivity, with corresponding *p* values of 0.034 and 0.008, respectively. Additionally, MRI temporal resolution greater than 5 s was found to be associated with greater specificity (*p* = 0.037), while studies involving more than 110 lesions were associated with a higher DOR with a *p* value of 0.021.

#### Individual UF-DCE MRI parameter analysis—MS vs TTE

**Fig. 4:**
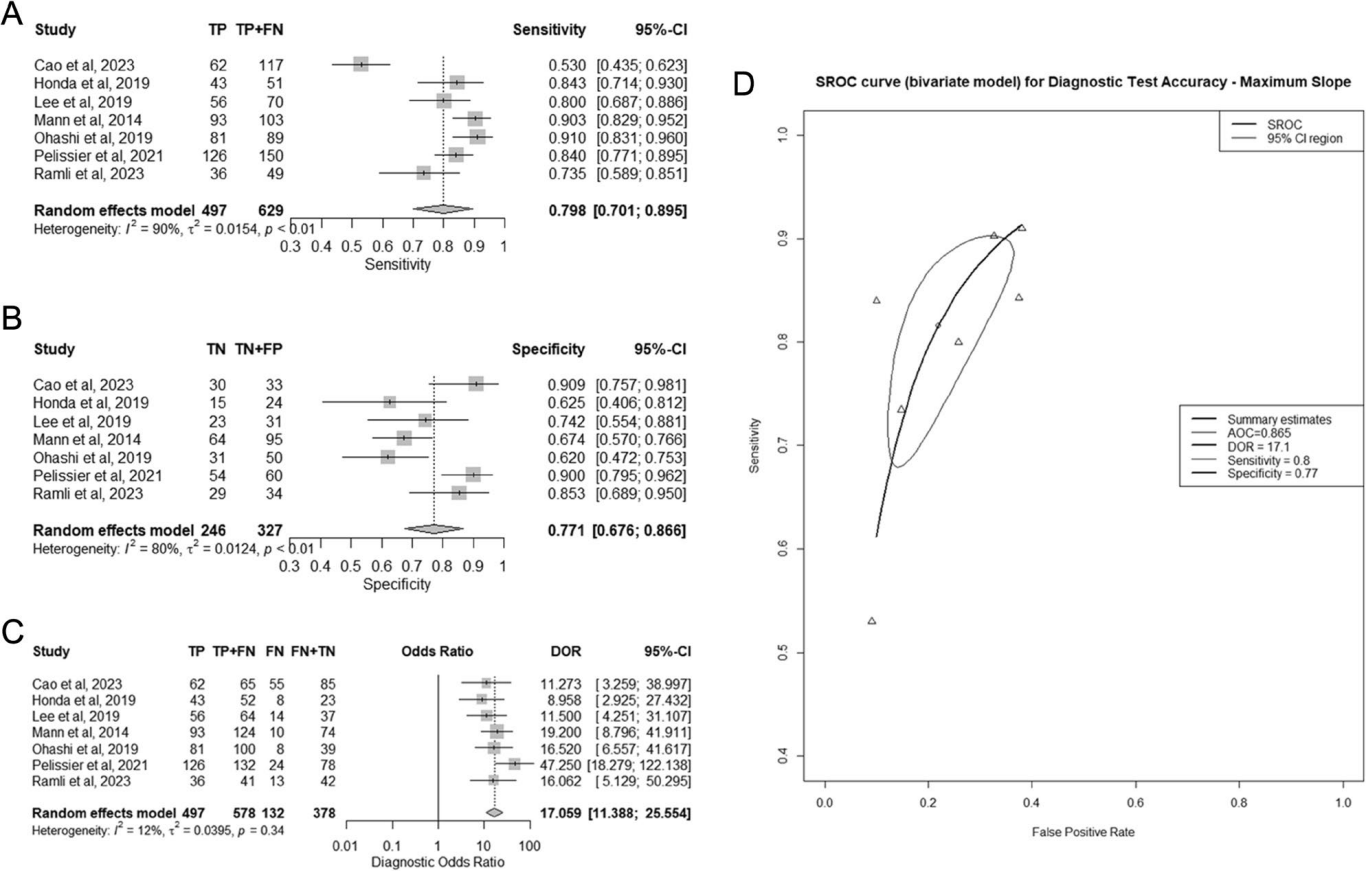
Diagnostic performance of maximum slope (MS) individually for differentiating benign from malignant breast lesions. **A** Summary forest plot of sensitivity. **B** Summary forest plot of specificity. **C** Summary forest plot of diagnostic odds ratio (DOR). **D** Hierarchic summary operating characteristics (SROC) curve

Seven studies incorporating 956 lesions were included in the MS meta-analysis [16, 28, 30, 32, 34, 35, 37] (Fig. 4A–D). The pooled sensitivity was 80% (95% CI 70–90%), the pooled specificity was 77% (95% CI 68–89%), the pooled DOR was 17.1 (95% CI 11.4–25.6), and the AUC of the SROC curve was 0.865 (95% CI 0.805–0.891).

**Fig. 5:**
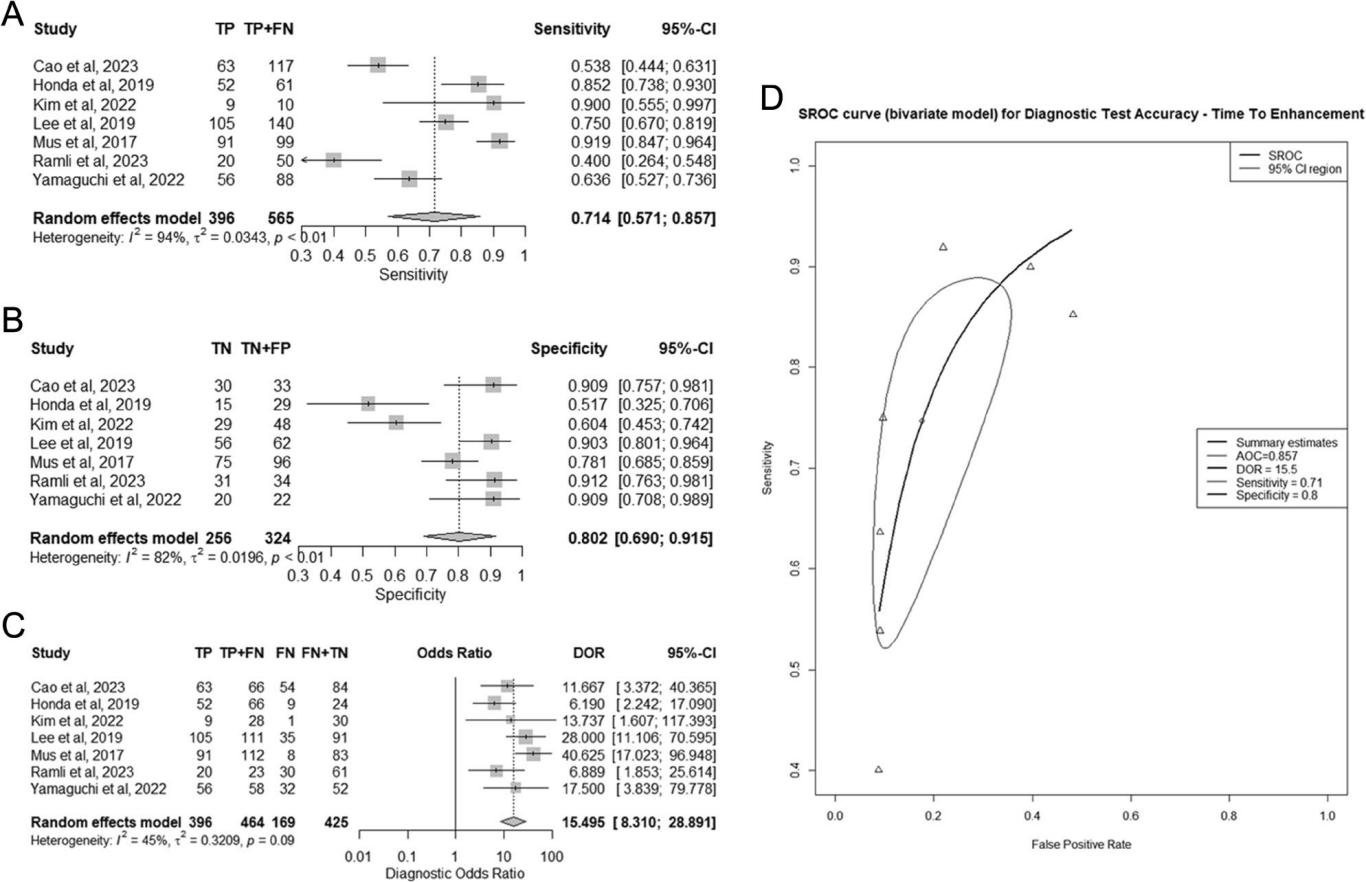
Diagnostic performance of time to enhancement (TTE) individually for differentiating benign from malignant breast lesions. **A** Summary forest plot of sensitivity. **B** Summary forest plot of specificity. **C** Summary forest plot of diagnostic odds ratio (DOR). **D** Hierarchic summary operating characteristics (SROC) curve

Seven studies comprising a total of 889 lesions were included in the TTE meta-analysis [17, 28, 30–32, 37, 39] (Fig. 5A–D). The pooled sensitivity was 71% (95% CI 57–86%), the pooled specificity was 80% (95% CI 69–82%), the pooled DOR was 15.5 (95% CI 8.3–28.9), and the AUC of the SROC curve was 0.857 (95% CI 0.763–0.889).

For both the MS and TTE analysis, there was a high risk for substantial heterogeneity for sensitivity and specificity, and a low risk of substantial variability for DOR (Figs. 4A–C, 5A–C). The correlation coefficients between sensitivity and specificity were −0.77 and −0.75 for MS and TTE respectively, suggesting low heterogeneity of the SROC curves.

In the “head-to-head” meta-regression analysis, as presented in Table 4S, there were no statistically significant differences observed between MS and TTE in terms of sensitivity, specificity, or DOR, with corresponding *p* values of 0.346, 0.638, and 0.880, respectively.

Table 2 provides an overview of various models along with their corresponding pooled accuracy measurements.

### Publication bias

The shape of the funnel plot is generally symmetrical, which indicates low publication bias (Figure 1S). Egger’s test also supports low publication bias (*p* = 0.585).

## Discussion

UF-DCE MRI is a promising new technique which has the potential to obtain kinetic information while reducing scan time. To the best of our knowledge, this is the first meta-analysis exploring the diagnostic accuracy of this novel approach. This comprehensive meta-analysis aggregated 16 studies and 2090 lesions to investigate the discriminatory power of UF-DCE MRI in differentiating benign from malignant breast lesions.

We found that when using UF-DCE MRI kinetic parameters, the pooled diagnostic sensitivity, specificity, DOR, and area under SROC for differentiating benign from malignant lesions are 83% (95% CI 79–88%), 77% (95% CI 72–83%), 18.9 (95% CI 13.7–26.2), and 0.876 (95% CI 0.83–0.887), respectively. Prior meta-analyses assessing the diagnostic accuracy full protocol DCE-MRI have found a pooled sensitivity, specificity, DOR, and area under the SROC of 87–93%, 74–85%, 18.8–91, and 0.86–0.96 respectively [41–43]. Except for sensitivity, which is slightly lower, our pooled figures fall within the range of these values, which is highly encouraging. Our work focused on the diagnostic accuracy of UF-DCE MRI as a stand-alone technique. In contrast, the previous abovementioned meta-analyses [41–43] examined the diagnostic accuracy of full-protocol breast MRI, which encompasses morphological data, kinetic curve data, and diffusion sequences. It is probable that by combining UF-DCE MRI with DCE-MRI sequences, we can expect an additive effect that will further enhance diagnostic accuracy. Many studies included in this meta-analysis lend support to this hypothesis. A few works have shown that the addition of UF-DCE MRI to DCE-MRI increased the diagnostic accuracy compared to DCE-MRI alone [29, 31, 32]. Other works have shown that UF-DCE MRI as a stand-alone technique is as good or even better than standard curve type analysis [17, 20, 30, 33, 34]. This suggests the possibility of reducing scan time while maintaining or potentially enhancing diagnostic accuracy. An option to consider involves integrating AB-MRI and UF-DCE MRI, allowing the utilization of kinetic data while simultaneously reducing examination time by eliminating the requirement for delayed scans. Researchers are encouraged to conduct more in-depth examinations in this direction in their future work.

In our analysis, we observed a considerable degree of heterogeneity in sensitivity and specificity assessments, while there was comparatively lower heterogeneity in the analysis of DOR and SROC. This outcome aligns with the conventional expectation of a negative correlation between sensitivity and specificity. As one of these values increases, the other tends to decrease, resulting in what is known as the “threshold effect” [44], which likely contributed to variations in heterogeneity. We used the random-effects model to enable pooling of the results within this context. For the DOR and SROC analysis, which provide a single measure merging the results of each diagnostic study thereby eliminating the “threshold effect,” heterogeneity was low.

We investigated additional sources of heterogeneity through a meta-regression analysis. Studies of larger scale that utilized Siemens MRI systems, featuring a temporal resolution exceeding 5 s and including younger women, tended to yield slightly better accuracy. The correlation between different technical parameters and diagnostic performance is particularly important, as there is no standardized protocol for UF-DCE MRI; each vendor and institution employ different MRI sequences. However, our findings were inconsistent across various analyses, making it challenging to draw firm conclusions. With the accumulation of more data, it is expected that a more standardized UF-DCE MRI sequence with optimal technical parameters can be established.

MS and TTE are the two most frequently utilized kinetic parameters in UF-DCE MRI, and most studies within this meta-analysis incorporated either one or both parameters. Based on our results, although MS achieved slightly higher accuracy than TTE individually, there is no statistically significant difference between the two methods. The parameters capture different kinetic values—MS is a measurement of the time-intensity curve and TTE measures the earliest time of lesion enhancement (Fig. 1). However, as both parameters reflect the same basic pathophysiological mechanism (AV shunting and capillary leakage), their yielding of similar outcomes is not unexpected. This observation might also explain why our meta-regression analysis failed to reveal any evidence supporting an increase in accuracy when using models combining more than one parameter, in contrast to our initial expectation. It is possible that these parameters are not additive but instead redundant.

In addition to its role in classifying breast lesions as benign or malignant, UF-DCE MRI may have other applications in the realm of breast cancer. These could include improving lesion conspicuity compared to DCE-MRI [45, 46], differentiating invasive breast carcinoma from ductal carcinoma in situ (DCIS) [30, 47, 48], predicting tumor prognostic markers and receptor status [29, 47–49], and predicting pathological complete response (pCR) after neoadjuvant treatment [50–52]. These potential roles warrant further exploration in upcoming dedicated systematic reviews and meta-analyses.

Our study had several limitations. First, many of the studies had suboptimal quality with regard to patient selection, index test, and reference standard. Second, we included only studies using UF-DCE MRI parameters exclusively, thereby limiting the number of available studies and affecting its statistical power. Third, there was significant variability among the included studies with respect to the kinetic parameter used in each study. This limitation is relevant specifically to our main analysis using the best model in each study. Nevertheless, as discussed previously, all kinetic parameters reflect a similar pathophysiological mechanism of rapid contrast leakage and AV shunting, resulting in faster wash-in within the first minute, and thus potentially allowing for pooling of the results. This is further supported by the low heterogeneity of DOR and SROC curves between the studies, the similar accuracy values of the two main kinetic parameters when used individually (MS and TTE), and the similar accuracy of models using one or multiple parameters. Furthermore, our analysis did not encompass studies utilizing artificial intelligence methods, primarily due to their scarcity. However, such an investigation could be undertaken in the future as more data become available. Finally, several of the studies did not report TP, FP, TN, and FN values directly, necessitating extraction of these values indirectly from the sensitivity, specificity, and total number of malignancies values, which may decrease accuracy.

In conclusion, based on our meta-analysis, UF-DCE MRI as a stand-alone technique has high accuracy in discriminating benign from malignant breast lesions. Our findings did not reveal a notable distinction in accuracy between the two primary UF-DCE MRI kinetic parameters, MS and TTE. We recommend further research, particularly focusing on the utilization of AI techniques and the integration of UF-DCE MRI with DCE-MRI sequences, to enhance diagnostic precision.

## Supplementary Information

Below is the link to the electronic supplementary material.

Supplementary file1 (PDF 246 KB)

## Abbreviations

AB-MRI: Abbreviated breast MRI
AI: Artificial intelligence
AUC: Area under curve
CI: Confidence interval
CS: Compressed sensing
DCE-MRI: Dynamic contrast-enhanced magnetic resonance imaging
DOR: Diagnostic odds ratio
FN: False-negative
FP: False-positive
KAUC: Kinetic area under the curve
MS: Maximum slope
PI: Parallel imaging
QUADAS**-**2: Quality Assessment of Diagnostic Accuracy Studies-2
SROC: Hierarchic summary operating characteristics
TN: True-negative
TP: True-positive
TTE: Time to enhancement
UF**-**DCE: MRI ultrafast MRI
VS: View sharing

## Declarations

### Guarantor

The scientific guarantor of this publication is Dr. Yoav Amitai.

### Statistics and biometry

No complex statistical methods were necessary for this paper.

### Study subjects or cohorts overlap

Some study subjects or cohorts have been previously reported because it is a meta-analysis.

### Methodology

• review and meta-analysis
