# Supplementary material for: The diagnostic performance of ultrafast MRI to differentiate benign from malignant breast lesions: a systematic review and meta-analysis
Source: Eur Radiol. 2024 Mar 21;34(10):6285–95. doi: 10.1007/s00330-024-10690-y (PMC11399157; doi:10.1007/s00330-024-10690-y)

The diagnostic performance of ultrafast MRI to differentiate benign from malignant breast lesions: A Systematic Review and Meta-Analysis

ELECTRONIC SUPPLEMENTARY MATERIAL

Table 1S Literature Search

Medline

breast cancer[MeSH Terms] OR breast[Title/Abstract] OR breast neoplasm[MeSH Terms] OR breast neoplasms[MeSH Terms]

AND

(ultrafast[Title/Abstract] OR ultra-fast[Title/Abstract] OR ultra fast[Title/Abstract])

AND

MRI[Title/Abstract] OR magnetic resonance [Title/Abstract] OR magnetic resonance imaging [MeSH Terms]

Embase

'nuclear magnetic resonance imaging'/exp OR

'mri':ab,ti OR 'magnetic resonance':ab,ti OR 'mri

scanner'/exp

AND

'ultrafast':ab,ti OR 'ultra fast':ab,ti OR

'ultra-fast':ab,ti

AND

breast:ab,ti OR 'breast disease'/exp OR 'breast

tumor'/exp

Cochrane

#1 MeSH descriptor: [Magnetic Resonance Imaging] explode all trees

#2 (magnetic resonance):ti,ab,kw

#3 MeSH descriptor: [Breast Neoplasms] explode all trees

#4 (breast):ti,ab,kw

#5 (ultrafast):ti,ab,kw

#6 (ultra fast):ti,ab,kw

#7 (ultra-fast):ti,ab,kw

#8 (#1 OR #2) AND (#3 OR #4) AND (#5 OR #6 OR #7)

Table 2S risk of bias summary

Study	RISK OF BIAS			
	PATIENT SELECTION	INDEX TEST	REFERENCE STANDARD	FLOW AND TIMING
**Abe et al**	☹	☺	☹	☺
**Cao et al**	☺	☺	☺	☺
**Goto et al**	☺	☹	☹	☺
**Honda et al**	?	☺	☺	☺
**Kim et al**	☹	☺	☹	☺
**Lee et al**	☹	☺	☹	☺
**Mann et al**	☺	☺	☹	☺
**Mori et al**	☹	☺	☹	☺
**Mus et al**	☺	☺	☹	☺
**Ohashi et al**	?	?	☹	☺
**Onishi et al**	☺	☹	☺	☺
**Pelissier et al**	☹	?	☹	☺
**Peter et al**	?	☺	☹	☺
**Ramli et al**	☺	☺	☺	☺
**Van zeist et al**	☺	☺	☺	☺
**Yamaguchi et al**	?	?	☹	☹

☺Low Risk ☹High Risk ? Unclear Risk

Table 3S: Multivariate meta-regression models evaluating the impact of different covariates on sensitivity, specificity and DOR for the main analysis

Sensitivity

**Covariate**	**Beta coefficient (95% CI)**	**Standard error**	***p*-value**
Number of lesions: >110 – reference <110	0 (-0.1;0.1)	0.051	0.994
Mean age	-0.017 (-0.032-;-0.001)	0.008	**0.034**
Cancer prevalence: >60% - reference<60%	0.003 (-0.094;0.101)	0.05	0.952
MRI system: Philips – reference Siemens<br>GE – reference Siemens	0.018 (-0.0714;0.107)<br>-0.165 (-0.287;-0.043)	0.046<br>0.062	0.694<br>**0.008**
Temporal resolution: >5sec – reference < 5sec	-0.053 (-0.147;0.041)	0.048	0.27
Kinetic threshold decided: a priori – reference post hoc	0.062 (-0.046;0.171)	0.055	0.262
Reference standard: Histopathology or FU – reference only histopathology	0.045 (-0.059;0.15)	0.053	0.397
Number of readers: more than 1 per lesion – reference only 1 per lesion	-0.026 (-0.121;0.069)	0.048	0.59
Kinetic parameter: combinations of parameters – reference single parameter	-0.012 (-0.136;0.111)	0.063	0.846
Acceleration method: CS – reference PI<br>VS – reference PI	-0.037 (-0.185;0.11)<br>-0.01 (-0.135;0.115)	0.075<br>0.063	0.621<br>0.876
Injection speed: >2ml/sec – reference ≤2ml/sec	-0.02 (-0.122;0.077)	0.051	0.658

Specificity

**Covariate**	**Beta coefficient (95% CI)**	**Standard error**	***p*-value**
Number of lesions: >110 – reference <110	0.114 (-0.003;0.231)	0.06	0.056
Mean age	0.004 (-0.020;0.028)	0.012	0.748
Cancer prevalence: >60% - reference<60%	0 (-0.121;0.121)	0.062	0.999

MRI system: Philips – reference Siemens<br>GE – reference Siemens	-0.099 (-0.203;0.0.004)<br>0.103 (-0.006;-0.212)	0.053<br>0.062	0.06<br>0.066
Temporal resolution: >5sec – reference < 5sec	0.112 (0.007;0.218)	0.054	**0.037**
Kinetic threshold decided: a priori – reference post hoc	-0.093 (-0.224;0.037)	0.067	0.161
Reference standard: Histopathology or FU – reference only histopathology	0.065 (-0.159;0.095)	0.065	0.617
Number of readers: more than 1 per lesion – reference only 1 per lesion	0.017 (-0.108;0.142)	0.064	0.788
Kinetic parameter: combinations of parameters – reference single parameter	0.069 (-0.081;0.218)	0.076	0.369
Acceleration method: CS – reference PI<br>VS – reference PI	0.063 (-0.124;0.251)<br>0.085 (-0.064;0.234)	0.096<br>0.076	0.506<br>0.265
Injection speed: >2ml/sec – reference ≤2ml/sec	0.06 (-0.063;0.184)	0.063	0.34

DOR

**Covariate**	**Beta coefficient (95% CI)**	**Standard error**	***p*-value**
Number of lesions: >110 – reference <110	0.737 (0.111;1.364)	0.320	**0.021**
Mean age	-0.075 (-0.211;0.062)	0.07	0.284
Cancer prevalence: >60% - reference<60%	0.038 (-0.641;0.718)	0.347	0.912
MRI system: Philips – reference Siemens<br>GE – reference Siemens	-0.257 (-1.018;0.503)<br>-0.37 (-1.252;0.511)	0.388<br>0.449	0.507<br>0.409
Temporal resolution: >5sec – reference < 5sec	0.132 (-0.559;0.822)	0.352	0.709
Kinetic threshold decided: a priori – reference post hoc	-0.037 (-0.832;0.758)	0.406	0.927
Reference standard: Histopathology or FU – reference only histopathology	0.403 (-0.274;1.081)	0.346	0.243
Number of readers: more than 1 per lesion – reference only 1 per lesion	0.186 (-0.493;0.864)	0.346	0.592
Kinetic parameter: combinations of parameters – reference single parameter	0.026 (-0.832;0.884)	0.438	0.952
Acceleration method: CS – reference PI<br>VS – reference PI	0.031 (-1.14;1.21)	0.56<br>0.492	0.959<br>0.583

	0.27 (-0.694;1.234)		
Injection speed: >2ml/sec – reference ≤2ml/sec	0.418 (-0.249;1.085)	0.34	0.22

CI: Confidence interval **CS**: Compressed sensing. **FU**: Follow-up **GE**: General-electric **MR**I: Magnetic resonance imaging. **PI**: Parallel imaging. **VS**: View sharing.

Table 4S: "Head to Head" Multivariate meta-regression models using MS and TTE as covariates for different effect measures, showing only the TTE vs MS coefficients

**Kinetic parameter: TTE – reference MS**	**Beta coefficient (95% CI)**	**Standard error**	***p*-value**
Sensitivity	-0.082 (-0.253;0.089)	0.087	0.346
Specificity	0.035 (-0.111;0.181)	0.074	0.638
DOR	-0.057 (-0.793;0.679)	0.376	0.880

CI: Confidence interval **DOR**: Diagnostic odds ratio **MS**: Maximum slope **TTE**: Time to enhancement

Figure 1S: Funnel plot to assess publication bias for diagnostic odds ratio (DOR).

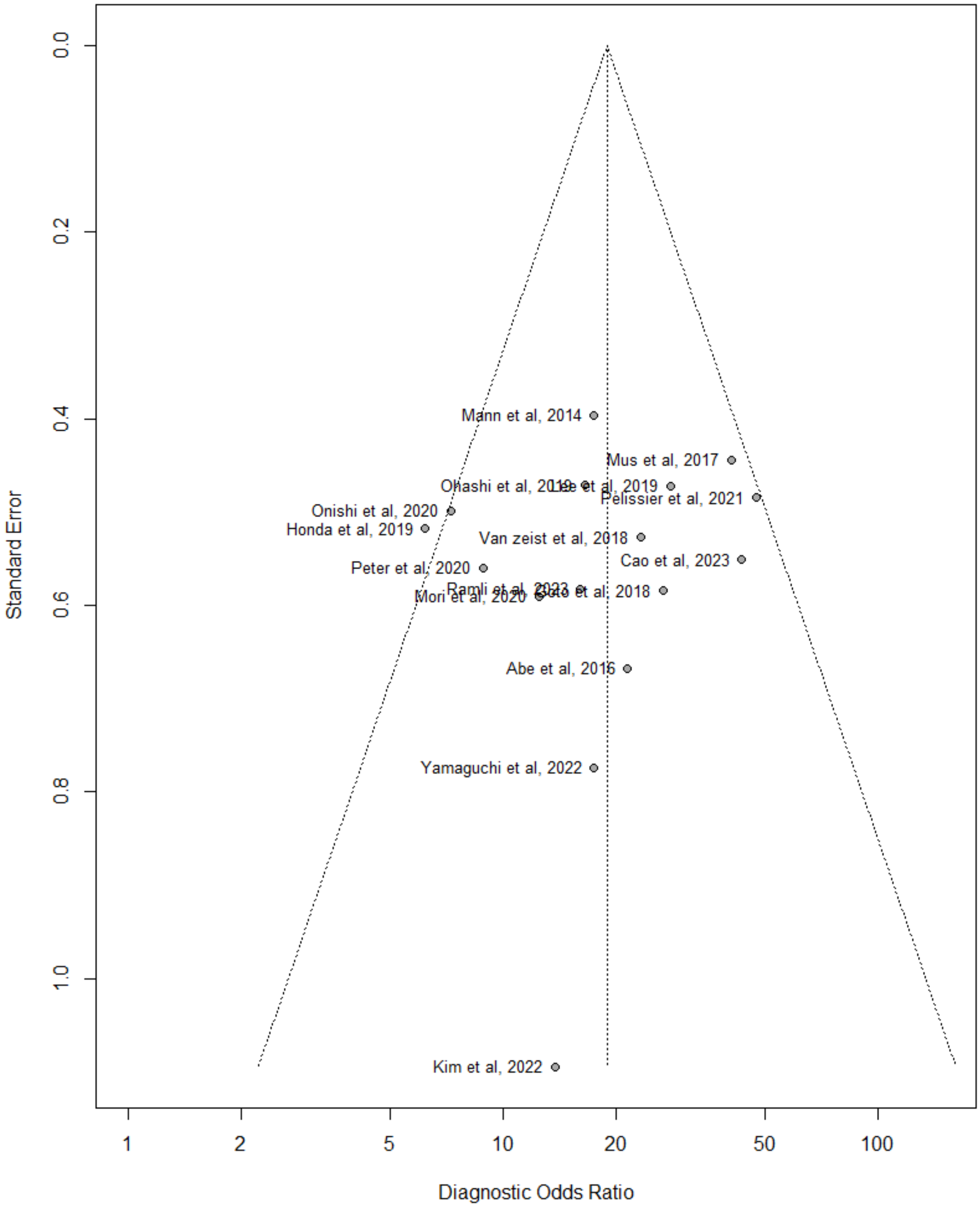

Supplement: Supplementary file 1 — Supplementary file1 (PDF 246 KB) [file 330_2024_10690_MOESM1_ESM.pdf]
